# Application of digital image correlation to study the activity and functional heterogeneity of the human temporalis muscle *in vivo* during unilateral chewing: a preliminary study

**DOI:** 10.3389/fbioe.2026.1767695

**Published:** 2026-04-21

**Authors:** Przemysław Stróżyk

**Affiliations:** Department of Mechanics, Materials and Biomedical Engineering, Wroclaw University of Science and Technology, Wroclaw, Poland

**Keywords:** human masticatory system, mandibular elevator muscles, mastication, muscle activity parameter, non-contact in vivo muscle activity examination, digital image correlation

## Abstract

**Introduction:**

This study aimed to demonstrate the usefulness of digital image correlation (DIC) for analysing the activity and heterogeneity of the temporalis muscle based on its displacements. While the standard measure for superficial muscles traditionally relies on muscle force determined via electrical biopotentials and numerical simulations, this paper presents results indicating that a kinematic parameter (muscle displacement) can effectively assess muscle function.

**Methods:**

The temporalis muscle was selected as the primary object of investigation, with *in vivo* measurements taken on both the working and non-working sides during unilateral chewing of three selected foods. The study group consisted of 8 healthy adult males aged 36.6 ± 9.4 years (BMI: 24.7 ± 0.8 kg/m^2^). A Dantec Q400 non-contact measurement system based on DIC was utilised. Reference images were captured at the mandible’s physiological resting position. The technique enabled continuous full-field mapping across the skin surface, while the final analysis was precisely confined to the area corresponding to the muscle fibres.

**Results and Discussion:**

The study demonstrated that the temporalis muscle’s functioning is complex and structurally heterogeneous. Specifically, the analysis revealed two distinct kinematic patterns: an asymmetric cycle (AC) in the anterior and middle parts responsible for force generation, and a fluctuating cycle (FC) in the posterior part serving as a dynamic stabiliser for the temporomandibular joint (TMJ). Furthermore, muscle kinematics were significantly modulated by both the chewing side and food texture. Spatial activation patterns suggest a task-specific biomechanical strategy, where the working side presses the mandible, while the non-working side stabilises it against excessive rotation. Ultimately, the results confirm that skin surface displacement can serve as a reliable, high-resolution measure of temporalis muscle activity.

## 1 Introduction

The development of modern biomedical engineering and experimental mechanics constantly strives for precise measurement and mathematical description of complex phenomena occurring in biological systems. Achieving high research efficiency requires appropriate measurement techniques. Currently, two commonly used approaches play a significant role in this area: electromyography (EMG) and numerical methods, such as the finite element method (FEM). The former is a diagnostic method that assesses muscle activity *in vivo* using bioelectrical impulses, while FEM is an *in silico* simulation method used for predictive analysis based on mathematical models. These methods are used to solve problems in two separate categories: biology and mechanics. Although EMG results can be used as input data for simulation, there is no direct link between the actual mechanical measurement and the digital model.

In this context, digital image correlation (DIC) appears to be an intermediate method that bridges the gap between EMG and FEM. Due to its measurement capabilities and full-field data analysis, DIC can serve as an integrated research system alongside the above-mentioned methods. In the long term, the combination of these techniques may lead to the creation of an advanced platform (DIC-HD-sEMG-FEM) that integrates *in vivo* measurements with *in silico* modelling. While DIC is widely established in the experimental mechanics of deformable bodies (Bambach et al., 2020; Barile et al., 2019; Pan et al., 2009) and *in vitro* biomechanics (La Barbera et al., 2021; Zwirner et al., 2021), its application in in vivo biomechanical studies remains scarce. However, preliminary work indicates its significant potential for analysing superficial muscles (Pachnicz and Stróżyk, 2023).

The temporalis muscle, a key element of the masticatory system, is characterised by a complex, fan-shaped fibre structure and an extensive origin (Hylander, 2006). In addition to generating forces, the temporalis muscle, together with the other masticatory muscles, actively participates in moving the mandible, e.g., during chewing, and also influences the stabilisation of the temporomandibular joint (TMJ) and the loads acting on the joint (Cuccia et al., 2011). These features determine its significant functional heterogeneity, which plays a critical role in healthy mandibular kinematics and is often altered in clinical conditions such as temporomandibular disorders (TMDs) (Zieliński et al., 2021; Woźniak et al., 2015; Szyszka-Sommerfeld et al., 2023). Individual parts of the muscle (the anterior part-vertical muscle fibres, the middle part-oblique muscle fibres, and the posterior part-horizontal muscle fibres) exhibit varied activity depending on the phase and method of mastication (Manns and Díaz, 1988; van Eijden et al., 1996; Stróżyk and Bałchanowski, 2023).

Traditional assessment methods based on point-by-point electromyography (EMG) measurements average the signal, thereby losing information about local dynamics. Attempts to determine muscle activity at high spatial resolution are currently based on high-density surface electromyography (HD-sEMG) (Rojas-Martínez et al., 2012; Cui et al., 2021; Xie et al., 2020; Thio-Pera et al., 2022). Although this method represents a significant advance in mapping two-dimensional bioelectrical signals, it has technical limitations due to the discrete nature of the multielectrode grid and the potential for large arrays to interfere with natural facial kinematics. Furthermore, to fully understand muscle biomechanics, it is necessary to consider not only bioelectrical activity but, above all, the mechanical response of tissues. According to the biomechanical principle of isovolumetric contraction, shortening of muscle fibres is accompanied by their transverse expansion, known as “muscle bulging”. Surface displacement thus reflects morphological changes in the muscle belly and is closely related to intramuscular pressure (Sleboda and Roberts, 2020) and bite force generation (Raadsheer et al., 1999).

The selection of the methodology in this study was directly motivated by these anatomical and functional complexities of the temporalis muscle, which require a high-resolution, full-field approach to be fully understood. Consequently, a significant research gap exists in developing non-invasive, high-resolution techniques capable of mapping this full-field mechanical response during dynamic functional tasks. In this paper, we propose using digital image correlation (DIC) to address this gap. DIC analyses images by tracking changes in the pixel intensity distribution using a dense network of virtual sensors (subsets), enabling continuous full-field displacement mapping. This approach introduces a new methodological quality by providing high spatial resolution to visualise heterogeneity (muscle bulging), enabling the assessment of local displacement gradients between adjacent muscle parts, and ensuring non-contact measurement that does not interfere with natural tissue dynamics.

From the perspective of deformable body mechanics, although the strain tensor fully describes the body’s deformation state, in this study, displacement (specifically, the component perpendicular to the surface of the temporalis muscle) was chosen as the primary parameter. Displacement directly reflects muscle bulging, whereas strain is a derived quantity dependent on the displacement field. The main aim of this study was to perform *in vivo* functional assessment of the temporalis muscle using digital image correlation (DIC). The analysis focused on measuring displacements perpendicular to the skin surface (muscle bulging), treating this parameter as a kinematic proxy for muscle activity. The study also aimed to verify whether this measurement enables the identification of muscle heterogeneity and the assessment of chewing-cycle dynamics within defined anatomical subareas. Furthermore, it was proposed to extend the functional analysis to include dynamic parameters (maximum and minimum displacements, peak-to-peak displacement amplitude, mean displacement, and displacement amplitude) to define the shape of the chewing curve. Measurements were performed on both the working and non-working sides during unilateral chewing of foods with different textures (Farella et al., 2008; Blanksma and van Eijden, 1995; Na and Kang, 2002; Kimoto et al., 2000; Piancino et al., 2005).

## 2 Materials and methods

The temporalis muscle activity studies were conducted as part of a project (The influence of selected food parameters on temporalis muscle activity and mandibular angular displacement during unilateral and bilateral alternating chewing, carried out in 2023–2027) approved by the Committee for Research Ethics of Wrocław University of Science and Technology (Commission opinion, 2023). The basic measurements were performed at the Department of Mechanics, Materials Science and Biomedical Engineering of the Wrocław University of Science and Technology.

### 2.1 Legal and ethical aspects

All participants were provided with complete information about the purpose and course of the study and the measurement method used. In accordance with the recommendations of the ethics committee, each participant was informed that: (1) participation in the study was entirely voluntary, (2) the study could be discontinued at any time at the participant’s request, and (3) the participant could withdraw from the study without giving a reason and without penalty.

The study did not involve: (1) invasive techniques, (2) collection of biological samples, and (3) deliberate changes in human behaviour. The study did not address controversial issues. The study was not physically or mentally stressful for the participants. Only adults who gave voluntary and informed consent could participate in the study.

Before the start of the study, each participant received an individual identifier consisting of a letter and a number in accordance with the requirements of the General Data Protection Regulation (GDPR) (Regulation EU, 2016). Therefore, the results obtained from the measurements were not, are not and will not be linked to the person being tested and will not be subject to profiling.

### 2.2 Selection criteria

During the selection of participants (j), individuals who met the following requirements were selected for the study: (1) naturally bald or willing to have their hair cut at least on the temporalis muscle area, (2) normal occlusion, (3) full permanent dentition, excluding third molars, (4) no primary dental treatment in the last three years-orthodontics, orthognathic surgery or extensive restorative treatment, (5) no history of temporomandibular joint disorders, (6) no history of changes in the temporalis muscle affecting its proper functioning, and (7) no history of allergic symptoms to paints used during measurements.

The study group consisted of 8 healthy adult males (mean age: 36.6 ± 9.4 years; mean height: 1.79 ± 0.07 m; mean body weight: 80.5 ± 6.7 kg; mean BMI: 24.7 ± 0.8 kg/m^2^). The restriction to males was directly related to the technical requirements of the DIC method, which requires unrestricted optical access to the skin surface of the temporalis muscle (requiring a shaved head).

### 2.3 Study object

The temporalis muscle was selected as the primary object of this *in vivo* study due to its critical biomechanical role in the masticatory system and its complex functional nature. Consequently, digital image correlation (DIC) was chosen and applied as the optimal tool to investigate it. The rationale for using this full-field optical method to study the temporalis muscle is dictated by the fact that: (1) it exhibits significant structural and functional heterogeneity, i.e. different parts of it generate different values of muscle force, contraction, energy and peak power (Ackland et al., 2017; de Zee et al., 2007; Guo et al., 2021; Laskin et al., 2006; Stróżyk and Bałachowski, 2018; Stróżyk and Bałachowski, 2020; Stróżyk and Bałachowski, 2023; Stróżyk and Bałachowski, 2025; van Eijden et al., 1996), which requires a high-resolution mapping approach; (2) anatomically, it is the most substantial muscle within the head and simultaneously the largest muscle involved in chewing, making its global assessment crucial; (3) it allows easy access from the outside; (4) it is located in an area with little subcutaneous fat; and (5) the mechanical response (functioning) of the muscle can be directly and accurately observed through the skin tissue covering its surface.

### 2.4 Food products

Products from three main food groups were selected for the study: dairy products (Gouda cheese), fruits (apples), and sweets (gingerbread). The selected products represent two distinct structural categories.

1. Quasi-homogeneous and continuous structure: represented by apples and Gouda cheese.
2. Multi-layered, heterogeneous sandwich-type structure represented by two layers of gingerbread and one layer of jam; this is a composite material characterised by high heterogeneity and discontinuity (especially gingerbread).

As a result, the masticatory muscles (Dellow and Lund, 1971; Lund, 1991) adapt their activity (including the temporalis muscle) to the current functional demands dictated by the texture of the food (Fitts et al., 1991; Mioche et al., 1999; Quintero et al., 2013; Stróżyk and Bałchanowski, 2016; Stróżyk and Bałchanowski, 2018; Stróżyk and Bałchanowski, 2023).

All study participants accepted the selected products. The sample dimensions were standardised to allow participants to bite freely, taking at least one complete bite cycle according to their preference (Netter, 2014).

To ensure hygiene and standardisation, the samples (except for the gingerbread bar) were prepared immediately before the measurements by a research team member equipped with: (1) a protective suit, (2) nitrile gloves, and (3) a disposable protective mask.

### 2.5 Measurement procedure

General procedures for using digital image correlation in experimental mechanics of solids and deformable bodies have been known for at least 10–20 years (Bambach et al., 2020; Barile et al., 2019; Jerabek et al., 2010; Kosturek et al., 2020; Pan et al., 2009; Palanca et al., 2016; Reu et al., 2007; La Barbera et al., 2021; Zwirner et al., 2021; Sutton et al., 2009). However, very little information is available on the procedures used in biomechanics, especially in studies of surface muscle activity *in vivo*. Therefore, a checklist was first prepared to verify all the elements on which the research is based, i.e., food samples, participant preparation, painting equipment, test station configuration, image acquisition parameters, and data analysis. In addition, a proprietary measurement procedure (based on the principles provided by Dantec Dynamics, 2025) was developed, primarily to minimise errors related to: (1) preparing participants for measurements, (2) performing measurements, and (3) compiling results.

The measurement procedure consisted of three basic stages: (1) painting the skin surface, (2) preparing the experimental setup and performing measurements, and (3) compiling the results. In addition, it was agreed that: (1) prior to the study, each participant would undergo training aimed at accustoming the masticatory organs to continuous unilateral chewing of food on the right side of the mandible (the working side (*W*)), and (2) one measurement day would correspond to one food item. If the participant agreed to chew another food on the same day, the measurements were continued; if they refused, another date was set.

Both the checklist and the measurement procedure are basic guides to minimise errors and ensure repeatability of results.

#### 2.5.1 Preparing participants for measurements-stage I

Before the start of the study, each participant (*j*) was measured (GIMA FAT-1 skinfold calliper, medical device Class: I M, CE marked (GIMA, 2025)) for the amount of adipose tissue covering the temporalis muscle on the left and right sides of the head. Based on the measurements taken (5 measurement points on the left and right sides, three measurements at each point) and considering the results obtained for all participants, the mean fat tissue value was 7.9% ± 2.0%.

**FIGURE 1:**
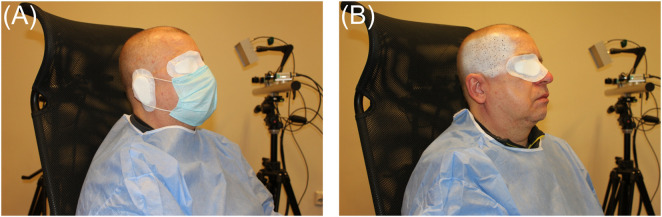
Preparation of the surface layer of skin tissue (epidermis) covering the temporalis muscle; **(A)** study participant with protective elements during painting, and **(B)** epidermis painted with white paint, on which black spots were randomly applied.

Measurements of temporalis muscle activity began with the preparation of the participant’s skin surface (epidermis). The surface had to be smooth (free of hair) and degreased; if it did not meet the requirements, the participant had to make corrections. Next, a stochastic pattern of high-contrast black and white spots was applied to the surface layer of the skin tissue (epidermis) covering the temporalis muscle. During the measurements, the pattern was used to determine displacements based on Istra 4D v4.6 × 64 software (Dantec Dynamics A/S, Skovlunde, Denmark). A standard precision painting technique based on airbrushing was used to prepare the random distribution of spots (Figure 1). Water-based paints were used for painting (Paint, 2025). During painting, the participant was asked to chew gum, which made it possible to reproduce the outline of the temporalis muscle.

In accordance with the ethics committee’s recommendation, each participant during painting (Figure 1) had to: (1) wear protective clothing, (2) cover their eyes and ears with a sterile dressing plaster (medical device), and (3) cover their nose and mouth with a disposable sterile mask.

#### 2.5.2 Experimental setup for DIC. Performing measurements-stage II

**FIGURE 2:**
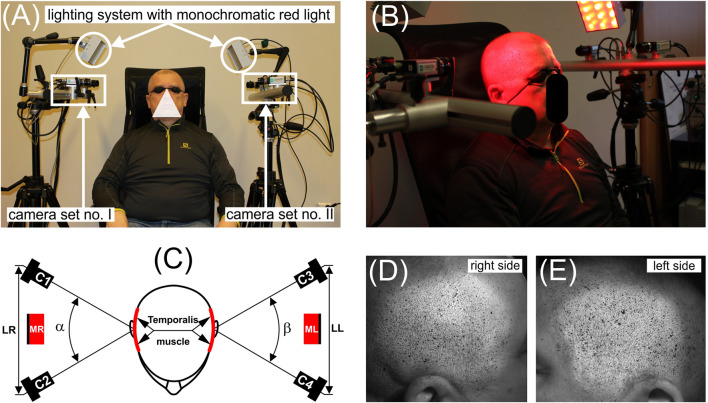
Experimental setup for DIC used to measure temporalis muscle activity during unilateral chewing of selected foods; **(A)** basic components of the measurement station, **(B)** participant illuminated by monochromatic red light with protective glasses, **(C)** camera and lighting positioning relative to the temporalis muscles, **(D)** and **(E)** example of a stochastic pattern of black and white spots generated by Istra 4D v4.6 × 64 software (Dantec Dynamics A/S, Skovlunde, Denmark) for a single image (so-called live image view), for the right and left sides of the head, respectively. Labels: camera set no. I (C1 and C2), camera set no. II (C3 and C4), MR-lighting on the right side, ML-lighting on the left side. *α* and *β* stereoscopic angles, on the working side (*W*) and non-working side (*N*), respectively.

A measurement station was prepared for measuring temporalis muscle activity (Figure 2), based on the Dantec Q400 non-contact optical digital image correlation system (Dantec Dynamics A/S, Skovlunde, Denmark), consisting of: (1) two synchronised sets of cameras GigE. (4x DSM 15331 HR GigE Camera 5 Mpx, 15 fps, Sensor Size: 2/3″, C-mount, Lens-Schneider-Kreuznach Xenoplan 1.4/17) positioned on both sides of the head, (2) two monochromatic red light sources illuminating the skin area covering the temporalis muscle on the left and right sides of the head (l), and (3) a computer with Istra 4D v4.6 × 64 software designed to record images during unilateral chewing of selected foods (*i*).

The measurement sensitivity of the described system is 1 µm in a 100 mm field of view. Thanks to the above parameters, the system’s displacement detection accuracy is 0.001% of the measurement range. Such high sensitivity is characteristic of high-end systems that require precise calibration and system stability.

Before proceeding with the calibration of the measurement system and measurements, the following was checked: (1) the cameras and light source were rigidly mounted on the tripod, (2) the position of the cameras relative to each other (stereoscopic angle α and β (Figure 2)) and relative to the participant’s head was constant during calibration and during measurements, (3) the position of the tripods and chair on the floor was marked for each participant, (4) the light source provided uniform illumination of the entire measurement area, (5) all cameras were set to: (a) the same frame rate and (b) focus individually for each participant.

After the participant took their seat, each camera set (set no. I and set no. II) and lighting were individually adjusted (height and distance from the head) on the left and right sides to suit their height (Figure 2).

Before starting the measurements, each camera set was calibrated using the AI-15-BMB-9x9 calibration chart (Dantec Dynamics A/S, Skovlunde, Denmark), and then reference photos of the temporalis muscle were taken for each side of the head (*l*) and each food item (*i*). Calibration was performed before each series of measurements (one series of measurements = one product), and each time any part of the system was moved. Calibration was supervised by software.

Since there is no information in the literature on the position of the mandible for which a DIC reference image should be taken, it was therefore taken for the physiological resting position of the mandible (Tingey et al., 2001), characterised by minimal muscle contraction activity (Sun et al., 2022).

Data recording (images) was performed automatically by the DIC system after activating the “start recording” icon (Istra 4D v4.6 × 64 software) with the simultaneous “start” command (the participant began chewing a bite of food). The measurements were terminated upon a clear signal from the participant, i.e., after swallowing the bolus. Four measurements (*n*) were performed for each food item. The study used an image recording speed of 15 frames per second (15 fps).

Each participant sat comfortably and steadily during the measurements with their back against the chair’s backrest (Figure 2).

For safety reasons, participants had to wear red light filter glasses (Figure 2).

#### 2.5.3 Theoretical assumptions

Analysis of muscle activity based on displacement measurements is based on a simplified mechanical model defined by the following three fundamental assumptions.

1. Perfect adhesion: a state of perfect mechanical coupling at the interface of muscle, fascia, and skin.
2. Direct transmission: Based on the mean tissue fat value (7.9% ± 2.0%), it was assumed that the displacements of a point on the skin surface (x, y, z)_skin_ directly correspond to the displacements of the underlying muscle point (x, y, z)_muscle_. This assumption is based on the fundamental principles of deformable body mechanics, specifically those related to thin-walled structures (e.g., pressure vessels, thin shells, and sandwich structures). In such systems, transverse displacement is transferred across the thin wall with negligible attenuation. Based on the first and second assumptions, it can be inferred that there is no relative motion at the tissue interface during contraction of the temporalis muscle.

#### 2.5.4 Results processing-stage III

After the measurements were completed, the measurement data had to undergo a universal correlation evaluation for each measurement step to verify that the results had been recorded correctly. Based on the evaluation, a set of results was prepared, taking into account all steps, which was used to visualise the measurement data in the form of a 2D overlay (displacement) on the camera image.

The evaluation and visualisation were performed per the Software Manual Q-4xx System version. 2.8.4 in the Istra 4D v4.6 × 64 software (Dantec Dynamics A/S, Skovlunde, Denmark).

In many publications on the biomechanics of the masticatory system, particularly the mandibular elevator muscles, the temporalis muscle is divided into three parts (Hylander, 2006). Considering the possibility of digital image correlation (measurements and analysis of the entire muscle) and the heterogeneity of the temporalis muscle (van Eijden et al., 1996), it was decided that the muscle surface would be divided into a larger number of parts. The subdivision allows a more detailed analysis of the muscle activity than when using electromyography. Therefore, the standard procedure for processing results in typical experimental studies was extended to include requirements for introducing a non-standard division of the temporalis muscle. It was assumed that, on the muscle surface (in the working and non-working sides), an area corresponding to the external muscle fibres would first be introduced. Then, the area will be divided into subareas (circles) for which local coordinate systems will be defined. When processing the results, it was assumed that the subarea size would be defined based on the area with the highest activity, i.e., the area where muscle tension is highest during chewing (the most significant displacement). Since Istra 4D v4.6 × 64 software does not allow copying defined areas and subareas, they were created individually for each participant, each page, and each food item.

Based on the accepted muscle division, the mean values of displacements perpendicular to the surface of each subarea were determined. The data obtained was then exported to Microsoft Excel (MS Office 2021 Professional Plus) for standard statistical analysis.

#### 2.5.5 Minimising measurement errors

In order to reduce the impact of the errors on the measurement results, the measurement system was calibrated per the rules specified in the Software Manual Q-4xx System version. 2.8.4. In addition, during the tests, it was assumed that the measurement system had to be calibrated after each change of food and participant.

When processing the results, the Remove Rigid Body Movements (RBMR) option was used, i.e., head movement (motion artefacts) was eliminated. As a result, the final results concerned only the temporalis muscle.

Errors resulting from the so-called subjective evaluation of the researcher were minimised by introducing the rule that each stage of the measurement procedure was controlled and performed by only one team member.

The measuring devices and software introduced partial automation of measurements and evaluation of measurement data. Namely, the measurement system automatically took photos during the measurements. At the same time, the evaluation of the results was semi-automatic, i.e., the software evaluated the measurement results based on the parameters entered by the researcher, but the decision to qualify or reject them was made by the person responsible for this stage of the research.

The same research protocol and conditions were applied to all participants (the temperature in the laboratory room was 21 °C-environmental factor).

### 2.6 Data processing and classification

#### 2.6.1 Definition of regions of interest and division into subareas

The analysis of the results obtained from DIC required, first of all, the introduction of the so-called Regions of Interest (ROI), which were used to define the areas visible to the cameras on the working (*W*) and non-working (*N*) sides. Regions of Interest were introduced in the reference image and then automatically transferred to subsequent images.

**FIGURE 3:**
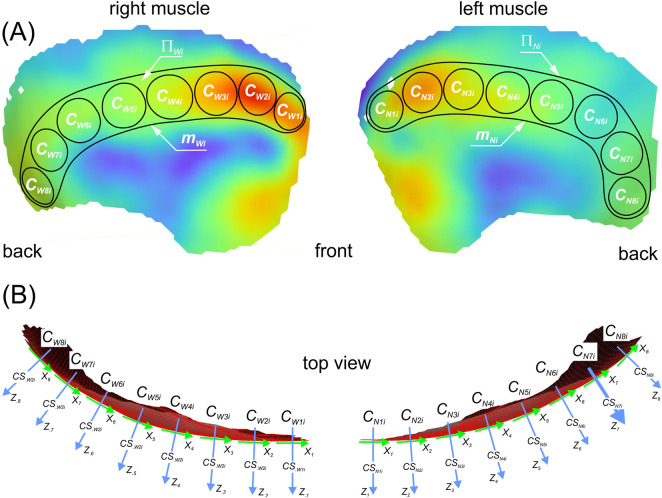
Diagram of the division of the temporalis muscle into regions (*Π*_*li*_), subareas (*C*_*lki*_) and the location of local coordinate systems (*CS*_*lki*_); **(A)** the displacement contours and **(B)** the curvature of the temporalis muscle. (*l* = *W*, *N*; *k* = 1, 2, … 7, 8; *i* = c, f, b).

Since the results do not allow for an unambiguous, classic division of the muscle into the anterior part (AT), the middle part (MT) and the posterior part (PT) (Hylander, 2006), therefore, based on its anatomical structure (Standring, 2004; Netter, 2006), areas corresponding to the surface muscle fibres on the working side (*Π*_*Wi*_) and the non-working side (*Π*_*Ni*_) were defined, bounded by the *m*_*Wi*_ and *m*_*Ni*_ lines (Figure 3A). Then, based on the subarea with the highest activity, a so-called circle pattern (*k*) was established, which was used to divide the muscle into subareas. Based on established individual patterns, the temporalis muscle of each participant was divided into 8 subareas to cover the muscle’s functional areas. The division was made on both the working and non-working sides. Taking all participants into account, the mean diameter of the circle was 19 ± 2.8 mm.

Due to the curvature of the temporalis muscle (Figure 3B), a local coordinate system (*CS*_*lki*_) was introduced for each circle in order to determine the mean displacement (*Z*_*lki*_) perpendicular to the surface of each circle. The *Z*_*lki*_ value was automatically determined based on virtual sensors (subsets) located within the circle.

The rationale for introducing 8 specific subareas, rather than relying solely on the classic division into three broad anatomical zones, was dictated by the preliminary analysis of the displacement fields. It revealed that averaging the high-resolution DIC data into only three regions would mask subtle, local kinematic gradients (e.g., distinct activation peaks) that are crucial for understanding the functional heterogeneity of the temporalis muscle.

For functional interpretation, it was assumed that each of the three anatomical parts of the temporalis muscle (Hylander, 2006) would correspond to selected subareas: the anterior part (*C*_*l1i*_, *C*_*l2i*_, *C*_*l3i*_), the middle part (*C*_*l4i*_, *C*_*l5i*_, *C*_*l6i*_), and the posterior part (*C*_*l7i*_, *C*_*l8i*_).

#### 2.6.2 Characterisation of chewing cycles

Since chewing is a cyclical process, specific parameters were calculated to define the cycle of displacements (*Z*_*lki*_) in individual subareas. Five basic parameters were determined for each participant (*P*_*1*_÷*P*_*8*_) using Equations 1–5: maximum displacement (*Z*_*lki,max*_), minimum displacement (*Z*_*lki,min*_), displacement amplitude (*Z*_*lki,a*_), mean displacement (*Z*_*lki,m*_), and the range of displacement changes (Δ*Z*_*lki*_).

$${\bar{Z}}_{lki,\max}=\frac{1}{4}\sum_{n=1}^{4}\left(Z_{lki,\max}\right)$$ (1)

$${\bar{Z}}_{lki,\min}=\frac{1}{4}\sum_{n=1}^{4}\left(Z_{lki,\min}\right)$$ (2)

$${\bar{Z}}_{lki,a}=\frac{1}{4}\sum_{n=1}^{4}\left[0.5\left(Z_{lki,\max}‐Z_{lki,\min}\right)\right]$$ (3)

$${\bar{Z}}_{lki,m}=\frac{1}{4}\sum_{n=1}^{4}\left[0.5\left(Z_{lki,\max}+Z_{lki,\min}\right)\right]$$ (4)

$$\Delta {\bar{Z}}_{lki}=\left({2\bar{Z}}_{lki,a}\right)$$ (5)

where:

*l* = *W*, *N*; *k* = 1, 2, … 6, 7, 8; *i* = *c*, *f*, *b*

Then, based on the results obtained for all participants (P1÷P8), the mean values of the above-mentioned parameters were calculated (Equations 6–10) and are given in Table 1 depending on the food (*i*), side of the mandible (*l*) and subarea (*C*_*lki*_).

$$\bar{X}Z_{lki,\max}=\frac{1}{8}\sum_{j=1}^{8}\left({\bar{Z}}_{lki,\max}\right)$$ (6)

$$\bar{X}Z_{lki,\min}=\frac{1}{8}\sum_{j=1}^{8}\left({\bar{Z}}_{lki,\min}\right)$$ (7)

$$\bar{X}Z_{lki,a}=\frac{1}{8}\sum_{j=1}^{8}\left({\bar{Z}}_{lki,a}\right)$$ (8)

$$\bar{X}Z_{lki,m}=\frac{1}{8}\sum_{j=1}^{8}\left({\bar{Z}}_{lki,m}\right)$$ (9)

$$\bar{X}\Delta Z_{lki}=2\bar{X}Z_{lki,a}$$ (10)

where:

**TABLE 1:** Average parameter values (maximum displacement ( $\bar{X}Z_{lki,\max}$ ), minimum displacement ( $\bar{X}Z_{lki,\min}$ ), displacement amplitude ( $\bar{X}Z_{lki,a}$ ), average displacement ( $\bar{X}Z_{lki,m}$ ), range of displacement changes ( $\bar{X}\Delta Z_{lki}$ ) characterising the temporalis muscle function cycle for selected subareas (*C*_*lki*_), depending on the working side (*W*) and non-working side (*N*) and selected foods (*i*). ±SD is given for each parameter. (*l* = *W*, *N*; *k* = 1, 2, … 7, 8; *i* = *c*, *p*, *b*).

Parameter [mm]	Muscle part							
	Anterior			Middle			Posterior	
	Subareas							
	***C***_***l1i***_	***C***_***l2i***_	***C***_***l3i***_	***C***_***l4i***_	***C***_***l5i***_	***C***_***l6i***_	***C***_***l7i***_	***C***_***l8i***_
Gouda Cheese								
working side								
$\bar{X}Z_{\text{Wkc},\max}$	0.84 ± 0.12	0.98 ± 0.13	0.68 ± 0.13	0.28 ± 0.08	0.11 ± 0.02	0.19 ± 0.03	0.34 ± 0.04	0.43 ± 0.07
$\bar{X}Z_{\text{Wkc},\min}$	-0.35 ± 0.06	-0.25 ± 0.06	-0.50 ± 0.09	-0.35 ± 0.05	-0.15 ± 0.03	0.06 ± 0.03	0.07 ± 0.02	0.10 ± 0.03
$\bar{X}Z_{\text{Wkc},m}$	0.25 ± 0.05	0.37 ± 0.04	0.09 ± 0.02	-0.04 ± 0.02	-0.02 ± 0.01	0.13 ± 0.03	0.21 ± 0.03	0.27 ± 0.05
$\bar{X}Z_{\text{Wkc},a}$	0.60 ± 0.08	0.61 ± 0.09	0.59 ± 0.11	0.31 ± 0.06	0.13 ± 0.02	0.06 ± 0.01	0.13 ± 0.02	0.16 ± 0.02
$\bar{X}∆Z_{\text{Wkc}}$	1.19 ± 0.16	1.23 ± 0.19	1.18 ± 0.22	0.63 ± 0.13	0.25 ± 0.05	0.13 ± 0.02	0.27 ± 0.04	0.33 ± 0.04
non-working side								
$\bar{X}Z_{\text{Nkc},\max}$	0.33 ± 0.06	0.55 ± 0.07	0.44 ± 0.07	0.26 ± 0.05	0.12 ± 0.03	0.26 ± 0.06	0.46 ± 0.07	0.61 ± 0.06
$\bar{X}Z_{\text{Nkc},\min}$	-0.74 ± 0.06	-0.39 ± 0.05	-0.43 ± 0.03	-0.37 ± 0.05	-0.16 ± 0.02	0.08 ± 0.02	0.15 ± 0.03	0.12 ± 0.03
$\bar{X}Z_{\text{Nkc},m}$	-0.20 ± 0.01	0.08 ± 0.03	0.01 ± 0.03	-0.06 ± 0.01	-0.02 ± 0.02	0.17 ± 0.04	0.31 ± 0.05	0.37 ± 0.04
$\bar{X}Z_{\text{Nkc},a}$	0.53 ± 0.06	0.47 ± 0.05	0.43 ± 0.04	0.32 ± 0.05	0.14 ± 0.02	0.09 ± 0.02	0.16 ± 0.02	0.25 ± 0.02
$\bar{X}∆Z_{\text{Nkc}}$	1.06 ± 0.12	0.93 ± 0.10	0.86 ± 0.09	0.63 ± 0.10	0.28 ± 0.04	0.19 ± 0.04	0.32 ± 0.05	0.5 ± 0.04
Apple								
working side								
$\bar{X}Z_{\text{Wkp},\max}$	0.89 ± 0.09	0.99 ± 0.09	0.76 ± 0.07	0.37 ± 0.07	0.34 ± 0.06	0.35 ± 0.07	0.45 ± 0.06	0.72 ± 0.06
$\bar{X}Z_{\text{Wkp},\min}$	-0.31 ± 0.03	-0.29 ± 0.03	-0.49 ± 0.05	-0.38 ± 0.04	-0.21 ± 0.04	0.16 ± 0.04	0.20 ± 0.05	0.32 ± 0.04
$\bar{X}Z_{\text{Wkp},m}$	0.29 ± 0.04	0.35 ± 0.05	0.14 ± 0.03	-0.01 ± 0.03	0.07 ± 0.02	0.26 ± 0.05	0.33 ± 0.06	0.52 ± 0.05
$\bar{X}Z_{\text{Wkp},a}$	0.60 ± 0.05	0.64 ± 0.04	0.63 ± 0.06	0.38 ± 0.05	0.27 ± 0.05	0.10 ± 0.03	0.13 ± 0.01	0.20 ± 0.02
$\bar{X}∆Z_{\text{Wkp}}$	1.20 ± 0.10	1.28 ± 0.08	1.25 ± 0.12	0.76 ± 0.10	0.54 ± 0.09	0.19 ± 0.05	0.25 ± 0.02	0.40 ± 0.03
non-working side								
$\bar{X}Z_{\text{Nkp},\max}$	0.68 ± 0.07	0.90 ± 0.08	0.47 ± 0.05	0.32 ± 0.05	0.08 ± 0.02	0.34 ± 0.06	0.77 ± 0.06	1.02 ± 0.07
$\bar{X}Z_{\text{Nkp},\min}$	-0.44 ± 0.04	-0.40 ± 0.07	-0.56 ± 0.03	-0.48 ± 0.02	-0.15 ± 0.01	0.22 ± 0.04	0.44 ± 0.08	0.44 ± 0.07
$\bar{X}Z_{\text{Nkp},m}$	0.12 ± 0.07	0.25 ± 0.06	-0.05 ± 0.02	-0.08 ± 0.02	-0.04 ± 0.02	0.28 ± 0.05	0.61 ± 0.07	0.73 ± 0.07
$\bar{X}Z_{\text{Nkp},a}$	0.56 ± 0.02	0.65 ± 0.03	0.52 ± 0.03	0.40 ± 0.03	0.12 ± 0.01	0.06 ± 0.02	0.17 ± 0.02	0.29 ± 0.01
$\bar{X}∆Z_{\text{Nkp}}$	1.12 ± 0.04	1.31 ± 0.05	1.04 ± 0.06	0.79 ± 0.06	0.23 ± 0.03	0.12 ± 0.03	0.33 ± 0.04	0.59 ± 0.01
Gingerbread Bar								
working side								
$\bar{X}Z_{\text{Wkb},\max}$	0.78 ± 0.06	0.88 ± 0.09	0.70 ± 0.05	0.31 ± 0.04	0.27 ± 0.03	0.38 ± 0.06	0.58 ± 0.06	0.65 ± 0.06
$\bar{X}Z_{\text{Wkb},\min}$	-0.49 ± 0.04	-0.40 ± 0.03	-0.60 ± 0.05	-0.50 ± 0.04	-0.15 ± 0.01	0.18 ± 0.03	0.28 ± 0.06	0.32 ± 0.04
$\bar{X}Z_{\text{Wkb},m}$	0.15 ± 0.01	0.24 ± 0.05	0.05 ± 0.03	-0.10 ± 0.01	0.06 ± 0.02	0.28 ± 0.04	0.43 ± 0.05	0.49 ± 0.04
$\bar{X}Z_{\text{Wkb},a}$	0.63 ± 0.05	0.64 ± 0.04	0.65 ± 0.03	0.41 ± 0.04	0.21 ± 0.02	0.10 ± 0.02	0.15 ± 0.02	0.33 ± 0.02
$\bar{X}∆Z_{\text{Wkb}}$	1.27 ± 0.10	1.28 ± 0.08	1.31 ± 0.07	0.82 ± 0.07	0.42 ± 0.04	0.20 ± 0.05	0.30 ± 0.03	0.66 ± 0.05
non-working side								
$\bar{X}Z_{\text{Nkb},\max}$	0.67 ± 0.07	0.78 ± 0.05	0.48 ± 0.06	0.41 ± 0.04	0.25 ± 0.05	0.18 ± 0.04	0.37 ± 0.05	0.75 ± 0.05
$\bar{X}Z_{\text{Nkb},\min}$	-0.51 ± 0.04	-0.45 ± 0.03	-0.58 ± 0.04	-0.50 ± 0.04	-0.13 ± 0.03	0.06 ± 0.01	0.23 ± 0.05	0.30 ± 0.06
$\bar{X}Z_{\text{Nkb},m}$	0.08 ± 0.03	0.17 ± 0.01	-0.05 ± 0.02	-0.05 ± 0.03	0.06 ± 0.02	0.12 ± 0.02	0.30 ± 0.05	0.53 ± 0.05
$\bar{X}Z_{\text{Nkb},a}$	0.59 ± 0.05	0.62 ± 0.04	0.53 ± 0.04	0.46 ± 0.03	0.19 ± 0.03	0.12 ± 0.02	0.07 ± 0.01	0.23 ± 0.02
$\bar{X}∆Z_{\text{Nkb}}$	1.18 ± 0.08	1.23 ± 0.05	1.06 ± 0.08	0.92 ± 0.06	0.39 ± 0.07	0.23 ± 0.04	0.15 ± 0.02	0.45 ± 0.04

*l* = *W*, *N*; *k* = 1, 2, … 6, 7, 8; *i* = *c*, *f*, *b*

A preliminary data analysis (Table 1) showed that the activity of the temporalis muscle in subareas (*C*_*Wki*_, *C*_*Nki*_) can be represented by two cycles, i.e. (1) asymmetric cycle (AC)-characterised by the fact that the mean values of maximum displacements ( $\bar{X}Z_{lki,\max}$ ) and minimum displacements ( $\bar{X}Z_{lki,\min}$ ) have positive and negative values, respectively and (2) fluctuating cycle (FC)-characterised by the fact that both the mean values of maximum displacements ( $\bar{X}Z_{lki,\max}$ ) and minimum displacements ( $\bar{X}Z_{lki,\min}$ ) have positive values.

#### 2.6.3 Percentage difference analysis

To quantify the observed regional disparities in temporalis muscle activity, an additional comparative analysis was carried out based on the percentage difference (Δ) determined from the mean values of maximum displacement. The difference was calculated using the following general equation (Equation 11):

$$\left|\Delta \left|=\left[2\left(A-B\right)/\left(A+B\right)\right]\right.\cdot 100\%\right.$$ (11)

where.

1. A > B, if values on the same side of the mandible are compared
2. A is the value on the working side, and B is on the non-working side, if the values on both sides of the mandible are compared

#### 2.6.4 Statistical analysis

Due to the relatively small sample size (n = 8) and the nature of the *in vivo* biomechanical data, non-parametric statistical methods were employed.

The Wilcoxon signed-rank test was used to assess functional asymmetry between the working (*W*) and non-working (*N*) sides. To determine the effect of food texture (cheese, apple, gingerbread bar) on muscle displacement, the Friedman test for repeated measures was applied. Significant results from the Friedman test were further analysed using Dunn’s post-hoc test with a Bonferroni correction to account for multiple comparisons. The significance level was set at *α* = *0.05*. All statistical analyses were performed in Microsoft Excel (MS Office 2021 Professional Plus) using built-in statistical functions.

## 3 Results

Based on the conducted experiments, the results enabled analysis of kinematic parameters (displacement perpendicular to the temporalis muscle surface). Furthermore, statistical analysis was performed to objectively verify that the observed variations in local muscle activity are not merely the result of random biological noise but constitute a reproducible, significant biomechanical response to both the chewing side and food texture.

### 3.1 Kinematic patterns of the temporalis muscle

Based on the accepted boundary conditions (resting position of the mandible and corresponding reference image of the temporalis muscle) and the data provided in Table 1 a preliminary analysis showed that the activity of the temporalis muscle in subareas (*C*_*Wki*_, *C*_*Nki*_) can be represented by two cycles, i.e., asymmetric cycles (AC) and fluctuating cycles (FC).

**FIGURE 4:**
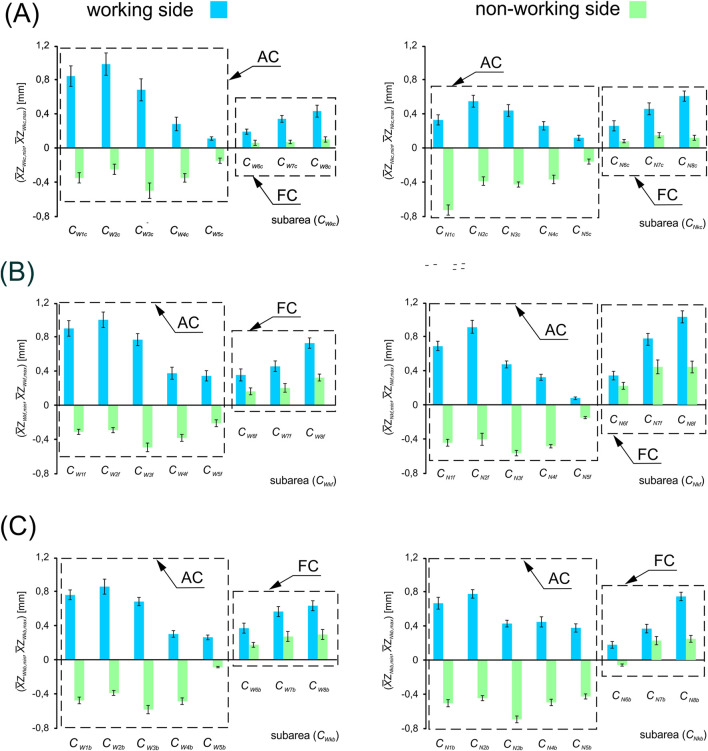
Mean values of the maximum displacement ( $\bar{X}Z_{Wki,\max}$ ; $\bar{X}Z_{Nki,\max}$ ) and minimum displacement ( $\bar{X}Z_{Wki,\min},$ ; $\bar{X}Z_{Nki,\min}$ ) for subareas (*C*_*Wki*_, *C*_*Nki*_), depending on the food being chewed (i) and the side of the mandible, i.e. the working side (*W*) and the non-working side (*N*). The dotted line marks the subareas of the temporalis muscle whose activity is represented by asymmetric cycles (AC) and fluctuating cycles (FC); **(A)** Gouda cheese, **(B)** apple and **(C)** gingerbread bar. (*k* = 1, 2, … 7, 8; *i* = *c, f, b*).

Figure 4 shows a graph based on the mean value of the maximum displacement ( $\bar{X}Z_{Wki,\max}$ ; $\bar{X}Z_{Nki,\max}$ ) and the mean value of the minimum displacement ( $\bar{X}Z_{Wki,\min}$ ; $\bar{X}Z_{Nki,\min}$ ) for subareas (*C*_*Wki*_, *C*_*Nki*_), depending on the food being chewed (*i*), respectively for the working side (*W*) and the non-working side (*N*).

**FIGURE 5:**
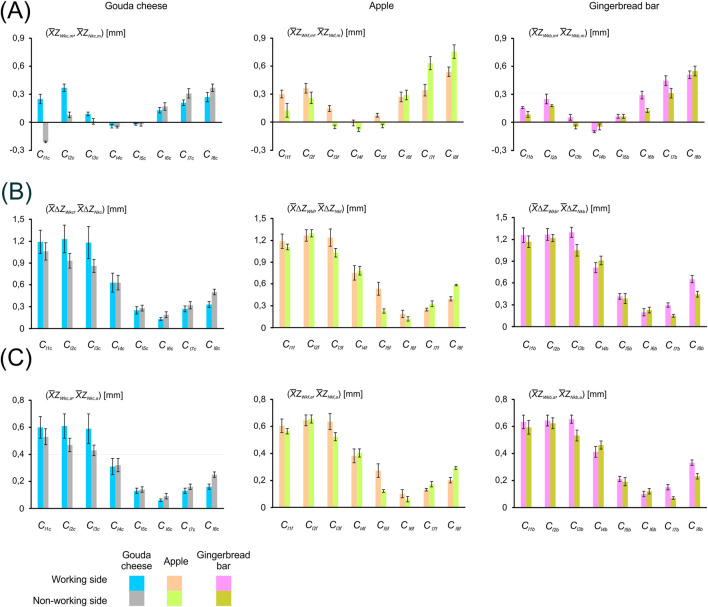
Graphical summary of mean values based on Table 1: **(A)** displacement values ( $\bar{X}Z_{lki,m}$ ), **(B)** peak-to-peak displacement amplitude ( $\bar{X}\Delta Z_{lki}$ ) and **(C)** displacement amplitude ( $\bar{X}Z_{lki,a}$ ) for temporalis muscle subareas, depending on the side of the mandible and the type of food chewed (Gouda cheese, apple, gingerbread bar). (*l* = *W*, *N*; *k* = 1, 2, … 7, 8).

Conversely, Figure 5 presents the mean values of displacement values ( $\bar{X}Z_{lki,m}$ ), mean values of peak-to-peak displacement amplitude ( $\bar{X}\Delta Z_{lki}$ ), and mean values of amplitude values ( $\bar{X}Z_{lki,a}$ ) for each subarea, depending on the side of the mandible (working and non-working) and the type of food. Furthermore, the data in both Figures 4, 5 are presented as box-and-whisker plots, where the whiskers indicate the standard deviation (±SD).

**FIGURE 6:**
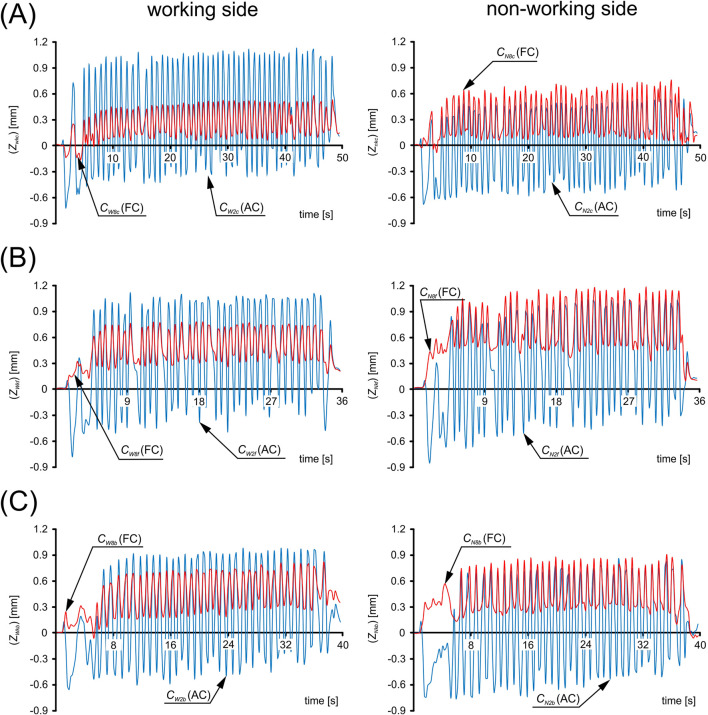
Example (participant P5) graphs of displacements (*Z*_*Wki*_, *Z*_*Nki*_) in the local coordinate system in the direction perpendicular to the surface of the subarea (*C*_*W2i*_ and *C*_*W8i*_) and (*C*_*N2i*_ and *C*_*N8i*_), corresponding to the maximum activity for a chewing cycle; **(A)** Gouda cheese, **(B)** apple and **(C)** gingerbread bar. (*k* = 1, 2, … 7, 8; *i* = *c, f, b*).

Figure 6 shows an example (participant P5) of graphs of displacements (*Z*_*Wki*_, *Z*_*Nki*_) for a chewing act, according to which the activity of the temporalis muscle subareas changed. The number of graphs was limited to two subareas (*C*_*W2i*_ and *C*_*W8i*_) and (*C*_*N2i*_ and *C*_*N8i*_), characterised by different types of cycles (AC and FC), depending on the food item (*i*) for the working side (*W*) and the non-working side (*N*), respectively.

### 3.2 Comparative analysis of subarea activity

Based on the established maximum displacement values, the percentage differences (*Δ*) between selected subareas were calculated to evaluate the functional heterogeneity of the temporalis muscle. The analysis was divided into two parts: determining the percentage difference between selected subareas on the same side of the mandible (working and non-working, respectively) and between corresponding subareas located on opposite sides of the mandible. The detailed results of these comparative calculations, categorized by food type, are presented in Table 2.

**TABLE 2:** Percentage differences (Δ*C*_*W(2–8)i*_ and Δ*C*_*N(2–8)i*_) between selected subareas located on the working side (*W*) only and on the non-working side (*N*) only, and the percentage difference (Δ*C*_*WN(2–2)i*_ and Δ*C*_*WN(8–8)i*_) between corresponding subareas located on opposite sides of the mandible, depending on the food (*i*). *i* = *c, f,* *b.*

Foods	Percentage difference between selected subareas			
	Side of mandible			
	Working	Non-working	Working vs. non-working	
	Δ*C*_*W(2-8)i*_ [%]	Δ*C*_*N(2-8)i*_ [%]	Δ*C*_*WN(2-2)i*_ [%]	Δ*C*_*WN(8-8)i*_ [%]
Gouda cheese (*c*)	78	10	35	56
Apple (*f*)	32	13	10	34
Gingerbread bar (*b*)	30	4	12	14

### 3.3 Spatial distribution of temporalis muscle displacement

To verify that the observed spatial variations in muscle displacement are physiologically robust and not due to random biological noise, a comprehensive statistical evaluation was conducted. This analysis mathematically validates the actual influence of both the mastication side and food texture on the localised kinematics of the temporalis muscle.

In order to accurately analyse the kinematic behaviour of the temporalis muscle, displacement amplitudes were assessed in eight subareas (*C*_*l1i*_-*C*_*l8i*_) for both the working side (*W*) and the non-working side (*N*). Statistical analysis confirmed that spatial activation patterns were asymmetrical and dependent on food texture (Tables 3 and 4).

**TABLE 3:** Mean values (standard deviation, ±SD) and statistical significance of differences between the working side (*W*) and the non-working side (*N*) in eight subareas (*C*_*l1i*_–*C*_*l8i*_) for Gouda cheese, apple, and gingerbread bar. Significance was determined using the Wilcoxon signed-rank test (*p < 0.05*). *l*= *W*, *N*; *i* = *c, f, b.*

Subareas	Gouda cheese (*c*) *W* vs *N* (*Mean* ± SD)	Statistical significance	Apple (*f*) *W* vs *N* (*Mean* ± SD)	Statistical significance	Gingerbread bar (*b*) *W* vs *N* (*Mean* ± SD)	Statistical significance
*C*_*l1i*_	*W*: 0.58 ± 0.09 *N*: 0.52 ± 0.06	p > 0.05 (ns/*)	*W*: 0.59 ± 0.05 *N*: 0.65 ± 0.03	*p < 0.05* (*N* > *W*)	*W*: 0.64 ± 0.05 *N*: 0.70 ± 0.03	*p < 0.05* (*N* > *W*)
*C*_*l2i*_	*W*: 0.61 ± 0.10 *N*: 0.47 ± 0.05	*p < 0.05* (*W* > *N*)	*W*: 0.64 ± 0.04 *N*: 0.56 ± 0.02	*p < 0.05* (*W* > *N*)	*W*: 0.61 ± 0.05 *N*: 0.63 ± 0.05	p > 0.05 (ns/*)
*C*_*l3i*_	*W*: 0.58 ± 0.12 *N*: 0.43 ± 0.05	*p < 0.05* (*W* > *N*)	*W*: 0.64 ± 0.06 *N*: 0.52 ± 0.03	*p < 0.05* (*W* > *N*)	*W*: 0.64 ± 0.04 *N*: 0.56 ± 0.03	*p < 0.05* (*W* > *N*)
*C*_*l4i*_	*W*: 0.31 ± 0.07 *N*: 0.32 ± 0.05	p > 0.05 (ns/*)	*W*: 0.38 ± 0.05 *N*: 0.41 ± 0.03	p > 0.05 (ns/*)	*W*: 0.40 ± 0.03 *N*: 0.48 ± 0.04	*p < 0.05* (*N* > *W*)
*C*_*l5i*_	*W*: 0.13 ± 0.02 *N*: 0.14 ± 0.01	p > 0.05 (ns/*)	*W*: 0.25 ± 0.05 *N*: 0.11 ± 0.01	*p < 0.05* (*W* > *N*)	*W*: 0.17 ± 0.02 *N*: 0.39 ± 0.03	*p < 0.05* (*N* > *W*)
*C*_*l6i*_	*W*: 0.07 ± 0.01 *N*: 0.09 ± 0.02	p > 0.05 (ns/*)	*W*: 0.10 ± 0.02 *N*: 0.06 ± 0.01	*p < 0.05* (*W* > *N*)	*W*: 0.10 ± 0.02 *N*: 0.10 ± 0.03	p > 0.05 (ns/*)
*C*_*l7i*_	*W*: 0.14 ± 0.02 *N*: 0.16 ± 0.02	p > 0.05 (ns/*)	*W*: 0.13 ± 0.01 *N*: 0.18 ± 0.02	*p < 0.05 (N > W*)	*W*: 0.14 ± 0.02 *N*: 0.07 ± 0.01	*p < 0.05* (*W* > *N*)
*C*_*l8i*_	*W*: 0.16 ± 0.02 *N*: 0.25 ± 0.02	*p < 0.05* (*N* > *W*)	*W*: 0.20 ± 0.02 *N*: 0.29 ± 0.01	*p < 0.05* (*N* > *W*)	*W*: 0.25 ± 0.02 *N*: 0.18 ± 0.02	*p < 0.05* (*W* > *N*)

/*(*ns*) - non-significant.

**TABLE 4:** The effect of food texture (Gouda cheese, apple, gingerbread bar) on the local displacement amplitude of the temporalis muscle. Statistical significance was determined using the Friedman test followed by Dunn-Bonferroni post-hoc analysis (*p < 0.05*).

Subareas	Gouda cheese (*c*)	Apple (*f*)	Gingerbread bar (*b*)	Statistical significance (test post-hoc)
Working side (*W*)				
*C*_*W1i*_	0.58 ± 0.09	0.59 ± 0.05	0.64 ± 0.05	*p < 0.05* (*b* > *c*)
*C*_*W2i*_	0.61 ± 0.10	0.64 ± 0.04	0.61 ± 0.05	*p > 0.05* (ns/*)
*C*_*W3i*_	0.58 ± 0.12	0.64 ± 0.06	0.64 ± 0.04	*p < 0.05* (*f* > *c*, *b* > *c*)
*C*_*W4i*_	0.31 ± 0.07	0.38 ± 0.05	0.40 ± 0.03	*p < 0.05* (*b* > *c*, *f* > *c*)
*C*_*W5i*_	0.13 ± 0.02	0.25 ± 0.05	0.17 ± 0.02	*p < 0.05* (*f* > *c*, *f* > *b*)
*C*_*W6i*_	0.07 ± 0.01	0.10 ± 0.02	0.10 ± 0.02	*p > 0.05* (*ns*/*)
*C*_*W7i*_	0.14 ± 0.02	0.13 ± 0.01	0.14 ± 0.02	*p > 0.05* (*ns*/*)
*C*_*W8i*_	0.16 ± 0.02	0.20 ± 0.02	0.25 ± 0.02	*p < 0.05* (*b* > *f*, *b* > *c*)
Non-working side (*N*)				
*C*_*N1i*_	0.52 ± 0.06	0.65 ± 0.03	0.70 ± 0.03	*p < 0.05* (*b* > c, *f* > *c*)
*C*_*N2i*_	0.47 ± 0.05	0.56 ± 0.02	0.63 ± 0.05	*p < 0.05* (*b* > c, *f* > *c*)
*C*_*N3i*_	0.43 ± 0.05	0.52 ± 0.03	0.56 ± 0.03	*p < 0.05* (*b* > c. *f* > *c*)
*C*_*N4i*_	0.32 ± 0.05	0.41 ± 0.03	0.48 ± 0.04	*p < 0.05* (*b* > *f*, *b* > *c*)
*C*_*N5i*_	0.14 ± 0.01	0.11 ± 0.01	0.39 ± 0.03	*p < 0.05* (*b* > *c*, *b* > *f*)
*C*_*N6i*_	0.09 ± 0.02	0.06 ± 0.01	0.10 ± 0.03	*p > 0.05* (*n*s/*)
*C*_*N7i*_	0.16 ± 0.02	0.18 ± 0.02	0.07 ± 0.01	*p < 0.05* (*f* > *b*, *c* > *b*)
*C*_*N8i*_	0.25 ± 0.02	0.29 ± 0.01	0.18 ± 0.02	*p < 0.05* (*f* > *c*, *f* > *b*)

/* (ns) - non-significant.

#### 3.3.1 Asymmetry between the working and non-working sides

The Wilcoxon test (Table 3) showed significant functional asymmetry between the working and non-working sides during chewing (*p* < *0.05*). For foods such as Gouda cheese and apple, the anterior part of the temporalis muscle (primarily *C*_*l2i*_, *C*_*l3i*_) showed significantly greater displacement on the working side, reflecting its primary role in the chewing phase. In contrast, in the middle part (*C*_*l4i*_-*C*_*l6i*_), activation levels decreased and evened out, showing no statistically significant differences between sides (*p* > *0.05*).

In the posterior part (*C*_*l8i*_), an unusual biomechanical phenomenon was observed. In the case of Gouda cheese and apples, the non-working side showed significantly greater displacement amplitudes than the working side (*p* < *0.05*). However, this compensatory mechanism was reversed entirely during gingerbread bar chewing, where the working side exhibited significantly greater displacement in the *C*_*l8i*_ subarea (*p* < *0.05*).

#### 3.3.2 The effect of food texture on muscle kinematics

To determine whether the observed differences in muscle displacement were random or directly related to the physical properties of the food piece, the Friedman test revealed (Table 4). The results clearly showed that food texture significantly alters the local kinematics of the temporalis muscle (*p < 0.05*).

The most significant changes were observed during gingerbread bar chewing. Compared to Gouda cheese and apple, gingerbread bar caused a statistically significant increase in displacement in the middle of the non-working side (*C*_*N5b*_), reaching peak values almost three times higher than those recorded for Gouda cheese. In addition, the apple required significantly more stabilising effort in the posterior part of the non-working side (*C*_*N5f*_) compared to the softer Gouda cheese (*p < 0.05*). These statistically significant differences confirm that the structural heterogeneity of the temporalis muscle enables it to adapt to the rheological requirements of different foods dynamically.

## 4 DISCUSSION

The presented study, using digital image correlation (DIC), provided novel *in vivo* data on the activity and functional heterogeneity of the temporalis muscle during unilateral chewing of selected foods. The analysis focused on kinematic patterns (displacement patterns) within defined subareas, demonstrating that the temporalis muscle is a specialised system whose activity is dynamically optimised depending on the side of mastication and the rheological properties of food.

### 4.1 Identification of functional muscle cycles: asymmetric cycle (AC) vs fluctuating cycle (FC)

Preliminary data analysis (Table 1) showed that temporalis muscle activity in the proposed sub-areas (Figure 3) can be represented by two distinct activity cycles (Figures 4, 6). The first group is represented by an asymmetric cycle (AC), while the second group corresponds to a fluctuating cycle (FC).

The results prove that the muscle fibres belonging to the anterior and middle subareas (*C*_*W(1–5)i*_ and *C*_*N(1–5)i*_) work according to the asymmetric cycle, while the posterior subareas (*C*_*W(6–8)i*_ and *C*_*N(6–8)i*_) function according to the fluctuating cycle. An analysis of the activity of the subareas functioning according to the asymmetric cycle on the working side (*W*) shows that the largest mean displacements ( $\bar{X}Z_{lki,\max}$ ) were recorded in *C*_*W2i*_, while the smallest were recorded in *C*_*W5i*_. Conversely, in the group working according to the fluctuating cycle, the highest activity was observed in *C*_*W8i*_, while the lowest was observed in *C*_*W6i*_ (Table 1). On the non-working side (*N*), displacements and cycles vary according to an identical distribution, with the difference that the values of the mean maximum ( $\bar{X}Z_{lki,\max}$ ) and minimum ( $\bar{X}Z_{lki,\min}$ ) displacements are predictably lower than on the working side.

The results also indicate that muscle fibres change their work cycle, regardless of mandibular side and food type. When analysing the values in Table 1 for subareas (*C*_*W5i*_ and *C*_*W6i*_) and (*C*_*N5i*_ and *C*_*N6i*_), it should be assumed, at this stage of the research, that this sudden change is a functional transition zone resulting from the adopted muscle division (8 subareas - Figure 3). Therefore, in future studies, it would be advisable to introduce a larger number of subareas, especially in areas where the work cycle undergoes sudden changes, to better reflect the local kinematic gradient. Furthermore, regarding these cycles, it is worth noting that adopting an initial mandibular position (reference photograph) other than that used in this study may significantly affect the baseline maximum and minimum displacement values. Consequently, such a change could affect the working cycle of the temporalis muscle subareas.

### 4.2 Functional heterogeneity: the interplay of mastication side and food texture

The integration of percentage-difference analysis with statistical testing provides evidence of temporalis muscle structural and functional heterogeneity. Our findings indicate that the highest activity is concentrated in the anterior and posterior subareas. For instance, the working side showed substantial activation disparities during Gouda cheese chewing (reaching 78% between the anterior and posterior parts-Table 2). In contrast, the middle part served as a functional transition zone where activation levels consistently evened out.

The observed greater mechanical activity (displacement) of the non-working side in the posterior part during consumption of Gouda cheese and apples carries significant biomechanical implications. It suggests that the horizontally oriented fibres on the non-working side act as active stabilisers of the temporomandibular joint (TMJ), preventing excessive joint loading during the unilateral crushing of these foods.

Interestingly, the reversal of this mechanism during gingerbread bar chewing highlights the muscle’s dynamic adaptation. Not only did the working side show higher displacement amplitudes in the posterior subarea, but the non-working middle part also experienced a nearly threefold increase in displacement compared to cheese. The gingerbread bar’s rheological properties demand a different stabilisation strategy, likely due to its higher viscosity or internal structure, which alters the effort required on the non-working side compared to apples. Based on our previous publications, the relationship between muscle contraction (i.e., muscle bulging) and muscle force is not linear during chewing; instead, it depends on the food’s mechanical properties. A linear relationship between force and displacement is expected for brittle materials, such as hard chocolate, whereas for other materials, linearity may hold only within a narrow range. These results confirm that the temporalis muscle is not a monolithic structure but a specialised system that dynamically optimises its activity patterns in response to the mechanical properties of the foods (Fitts et al., 1991; Mioche et al., 1999; Stróżyk and Bałachowski, 2018; 2023).

### 4.3 Full-field mechanical mapping (DIC) vs bioelectrical mapping (HD-sEMG)

The ability of DIC to detect such pronounced subarea differences aligns with recent advancements in neurophysiology, which increasingly recognise the non-uniform activation of motor units. Currently, attempts to determine such high-resolution muscle activity are dominated by high-density surface electromyography (HD-sEMG). The efficacy of modern HD-sEMG technology in capturing distinct local muscle excitations has been demonstrated across a range of complex applications, from evaluating spatially distributed activity in upper-limb muscles (Rojas-Martínez et al., 2012) and assessing neuromuscular coordination in professional piano players (Thio-Pera et al., 2022) to advanced spatial algorithms.

Although HD-sEMG provides a benchmark for other methods in two-dimensional bioelectrical signal mapping, it inherently measures the nerve impulse rather than the resulting mechanical signal. Dense multi-electrode arrays, while efficient, face physical limitations in facial applications, where sensor arrays can interfere with natural skin folds and reduce proprioception during dynamic tasks such as chewing. In this context, DIC provides a complementary, comprehensive kinematic equivalent to HD-sEMG. Using virtual subsets instead of physical electrodes, DIC provides non-contact, continuous mapping of the mechanical response (muscle bulge), effectively capturing the localised biomechanical consequences of a heterogeneous nerve impulse.

### 4.4 Limitations of the method and simplified model assumptions

It should be emphasised that the use of the DIC method for *in vivo* measurements is associated with specific technical and anatomical limitations.

The first key challenge is skin preparation. The precision of image correlation depends primarily on the quality of the stochastic pattern. The presence of hair causes decorrelation, necessitating shaving of the temporalis area. Additionally, during long sessions, the paint layer’s loss of elasticity can lead to microcracks, which the software may misinterpret as local strain concentration. This requirement was a significant recruitment barrier, resulting in a limited study group (n = 8) that was exclusively male. Nevertheless, the criteria adopted ensured the homogeneity of the study group, which was necessary to verify the feasibility and reproducibility of the proposed DIC methodology at this preliminary stage.

The second important aspect concerns functional anatomy and the adoption of a simplified biomechanical model. It must be acknowledged that DIC records skin surface deformations rather than direct muscle fibre activity. The skin, subcutaneous tissue, and temporalis fascia act as a mechanical filter, attenuating and smoothing the signal originating from the deeper muscle belly. Furthermore, the viscoelastic properties of the overlying soft tissue layers may introduce a slight phase lag or damping of the recorded mechanical response. Crucially, the central assumption that skin surface displacement is a reliable measure of underlying muscle activity was not directly validated through synchronous electromyography (EMG). Without such experimental validation, interpreting displacement magnitude as a direct physiological metric of muscle force remains an indirect approximation, functioning as a kinematic proxy rather than a direct force measurement.

In this preliminary stage, we adopted a simplified kinematic model where skin displacement directly reflects muscle bulging. However, from a biomechanical engineering perspective, determining muscle force from these displacements constitutes an inverse problem. Future iterations of this research will involve developing a validated mathematical model incorporating material nonlinearities, such as Mooney-Rivlin or Neo-Hookean hyperelastic constitutive equations, to translate DIC-mapped deformations into dynamic muscle loads more accurately.

Finally, the hardware parameters, in particular temporal resolution, pose a limitation. In this study, a recording speed of 15 fps was used. While this sampling density is sufficient to identify the maximum displacement amplitudes (AC and FC cycles) during a mean chewing frequency of 1.2–1.6 Hz (providing 9–12 frames per cycle), it may lead to an underestimation of rapidly changing phenomena during chewing. The use of high-speed cameras (e.g., >50 fps) in future studies will be necessary for more accurate kinetic analysis of muscle contraction.

Furthermore, while this study focuses on out-of-plane displacement as a primary indicator of muscle bulging, future research should incorporate the analysis of the strain tensor. Evaluating strain would provide a more normalised metric, allowing for a more comprehensive biomechanical characterisation of the muscle’s deformation state, independent of its absolute anatomical dimensions.

## 5 Conclusions

Based on the *in vivo* measurements using digital image correlation (DIC) and statistical analysis, the following conclusions can be drawn.

1. High-resolution functional mapping: The DIC method, combined with local subarea analysis, effectively reflects the high functional heterogeneity of the temporalis muscle. Assessing muscle activity based on classic anatomical parts (anterior, middle, and posterior) or a single averaged signal is insufficient, as it fails to reveal local displacement gradients. The proposed approach enables high-resolution mechanical mapping, offering more profound insights into muscle biomechanics beyond standard anatomical classifications.
2. Distinct functional cycles: The temporalis muscle operates through functionally distinct kinematic patterns. The anterior and middle parts work primarily in an asymmetric cycle (AC) dedicated to muscle force generation. In contrast, the posterior parts operate in a fluctuating cycle (FC), serving as a dynamic stabiliser for the mandible and temporomandibular joint (TMJ).
3. Dynamic adaptation to food texture: Muscle kinematics are significantly modulated by both the chewing side and food properties (*p < 0.05*). The spatial activation patterns suggest a task-specific biomechanical stabilisation strategy: on the working side, vertical fibres are primarily responsible for pressing the mandible against the maxilla, while on the non-working side, horizontal fibres are primarily responsible for stabilising the mandible against excessive rotation relative to the sagittal axis.
4. Methodological potential: As a preliminary proof of concept, DIC provides non-contact, full-field kinematic mapping that complements traditional electromyography (EMG). However, a direct quantitative comparison between a kinematic parameter (surface displacement) and a bioelectrical (EMG) or dynamic signal inherently requires the prior development and validation of an electromechanical mathematical model. Consequently, full methodological validation-coupling synchronous EMG-DIC acquisition with such a validated model-will be the focus of a subsequent study to establish DIC as a robust tool for clinical diagnostics and advanced computational models of the stomatognathic system.

## Data Availability

The datasets presented in this article are not readily available because the manuscript presents one of many possibilities for processing measurement data; therefore, the data will not be made available until the results have been fully processed and verified. Furthermore, in accordance with the guidelines of the Committee for Research Ethics of Wrocław University of Science and Technology, the data must undergo detailed verification to exclude the possibility of identifying project participants. Requests to access the datasets should be directed to przemyslaw.strozyk@pwr.edu.pl.
